# First Attempts of the Use of ^195^Pt NMR of Phenylbenzothiazole Complexes as Spectroscopic Technique for the Cancer Diagnosis

**DOI:** 10.3390/molecules24213970

**Published:** 2019-11-02

**Authors:** Bruna T. L. Pereira, Mateus A. Gonçalves, Daiana T. Mancini, Kamil Kuca, Teodorico C. Ramalho

**Affiliations:** 1 Department of Chemistry, Federal University of Lavras, P.O. Box 3037, Lavras 37200-00, MG, Brazil; bru.limapereira92@gmail.com (B.T.L.P.); mateus.a.g@hotmail.com (M.A.G.); daianateixeira60@yahoo.com.br (D.T.M.); 2 Department of Chemistry, Faculty of Science, University of Hradec Kralove, 500 02 Hradec Kralove, Czech Republic

**Keywords:** platinum complexes, ^195^Pt NMR, biological systems, cancer diagnosis

## Abstract

Platinum complexes have been studied for cancer treatment for several decades. Furthermore, another important platinum characteristic is related to its chemical shifts, in which some studies have shown that the ^195^Pt chemical shifts are very sensitive to the environment, coordination sphere, and oxidation state. Based on this relevant feature, Pt complexes can be proposed as potential probes for NMR spectroscopy, as the chemical shifts values will be different in different tissues (healthy and damaged) Therefore, in this paper, the main goal was to investigate the behavior of Pt chemical shifts in the different environments. Calculations were carried out in vacuum, implicit solvent, and inside the active site of P13K enzyme, which is related with breast cancer, using the density functional theory (DFT) method. Moreover, the investigation of platinum complexes with a selective moiety can contribute to early cancer diagnosis. Accordingly, the Pt complexes selected for this study presented a selective moiety, the 2-(4′aminophenyl)benzothiazole derivative. More specifically, two Pt complexes were used herein: One containing chlorine ligands and one containing water in place of chlorine. Some studies have shown that platinum complexes coordinated to chlorine atoms may suffer hydrolyses inside the cell due to the low chloride ion concentration. Thus, the same calculations were performed for both complexes. The results showed that both complexes presented different chemical shift values in the different proposed environments. Therefore, this paper shows that platinum complexes can be a potential probe in biological systems, and they should be studied not only for cancer treatment, but also for diagnosis.

## 1. Introduction

Cancer is currently one of the most discussed diseases, as it presents incredibly high mortality rates [1,2]. More specifically, cancer is a term employed for a group of diseases characterized by the acute growth of abnormal cells. Breast cancer is the class with the highest incidence in women, and it is associated with the high mortality rates in women with cancer [3,4,5].

Early-stage diagnosis is very important for cancer treatment, as it can reduce mortality rates and increase treatment possibilities [6,7,8]. Some imaging techniques are used as main approaches in cancer diagnosis, including mammography, magnetic resonance imaging (MRI), positron-emission tomography (PET), and computed tomography (CT). Furthermore, the use of these techniques with the help of biochemical biomarkers, such as proteins, could increase the chances of early diagnosis [3,9,10].

The use of imaging techniques associated with biomarkers as proteins, RNAs, microRNAs, and enzymes could also increase the chances of early diagnosis [3,11,12]. Furthermore, the use of compounds that have already been employed in medicine could potentially probe diseases [3,13,14]. In this context, Mavroid and colleagues synthesized the *cis-*dichloro(2′pyridinyl)methylamineplatinum(II) bonded to 2-(4′aminophenyl)benzothiazole derivative in order to study its properties as an antitumor agent [13].

This complex has two important moieties in cancer treatment: The platinum and the benzothiazole group. In 1965, the carcinogenic properties of the platinum group were discovered with the emergence of cisplatin, an important drug in chemotherapy [15,16,17]. Since then, different drugs using platinum has been developed to improve the treatment of different types of cancer [16,18,19]. Regarding the benzothiazole moiety, some studies have revealed that these types of molecules show potent and selective antitumor activity in vitro and in vivo against breast cancer cells, among other types of cancer [14,20,21].

**Figure 1:**
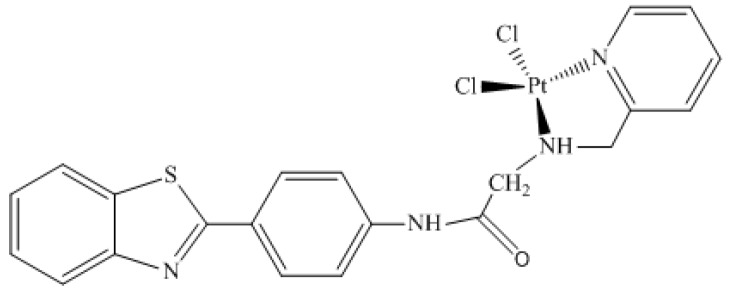
Cis-dichloro(2′pyridinyl)methylamineplatinum(II) bonded to 2-(4′aminophenyl)benzothiazole derivative (Complex 1).

**Figure 2:**
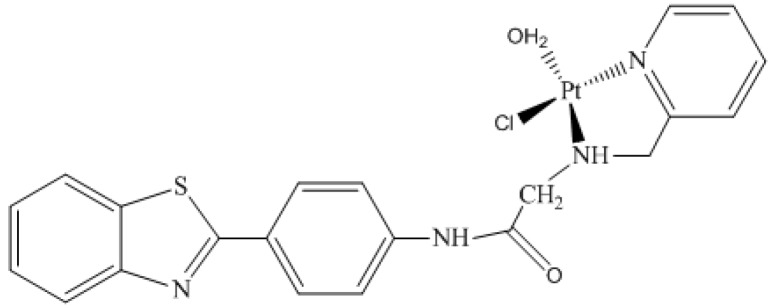
Monoaquated complex: Monoaquamonochloro(2′pyridinyl)methylamineplatinum(II) ion bonded to 2-(4′aminophenyl)benzothiazole derivative (Complex 2).

Accordingly, the purpose of this work was to use the *cis-*dichloro(2߰pyridinyl)methylamineplatinum(II) bonded to 2-(4′aminophenyl)benzothiazole derivative (Figure 1) as a potential probe in breast cancer diagnosis. It is also important to mention that this coordinated complex has already been investigated as a potential anticancer agent [13]. Furthermore, the strategy chosen for this work was to perform chemical shift analysis of ^195^Pt in different chemical environments (vacuum, implicit solvent, and inside active enzyme P13K), which was done through nuclear magnetic resonance (NMR) calculations. These calculations were performed for the monoaquated complex (Figure 2), as it is well-known that platinum complexes are hydrolyzed inside the cell [18,19,22]. Researcher groups have studied different platinum complexes with different ligands, as well as different oxidation numbers. Synthesis, characterization, and NMR analysis were also performed, and it was possible to see the sensitivity of the ^195^Pt chemical shift [23,24,25,26].

## 2. Results and Discussion

This discussion is divided into three main parts following the order established in the methodology section. Initially, it is worth mentioning that the validation step of the theoretical methodology was based on another work about NMR platinum complexes developed by Paschoal and collaborators (2017) [27]. In this research, the authors developed a basis set with relativistic correction for Pt(II) chemical shift calculations [27]. Moreover, for the validation step, we used two different methodologies for the platinum atom. In this step, optimization calculations were carried out at B3LYP/lanl2dz, B3LYP/aug-cc-pVTZ-pp, and Zora/B3LYP/Def2-TZVP levels, which is a relativistic method. The results are described in the supplementary material. In the second part, the ^195^Pt NMR chemical shift for complex 1 (Figure 1) was calculated in the gas phase, in solution, using implicit solvent (PCM) model, and in the P13K enzyme active site. In order to evaluate the thermal effects, the dynamic simulations were also carried out. In the last part, the same calculations were performed for the monoaquated complex (Figure 2).

For NMR chemical shift calculations, the notation was as follows: Level of shielding computation (PBEPBE)//level of geometry optimization (B3LYP) or MD simulation. For example, (PBEPBE//B3LYP) means shielding computation in vacuum//geometry optimization in vacuum, while (PBEPBE/PCM(H_2_O)//B3LYP/PCM(H_2_O)) means shielding computation with implicit solvent (PCM)//geometry optimization with implicit solvent (PCM). The same notation was employed when including the dynamic effect (ADMP). For example, (PBEPBE//ADMP) means shielding computation in vacuum//dynamic simulations in vacuum, while (PBEPBEPCM(H_2_O)//ADMP/PCM(H_2_O)) means shielding computation with implicit solvent (PCM)//dynamic simulations with implicit solvent (PCM).

### 2.1. Validation of ^195^Pt NMR Chemical Shifts Theoretical Methodology 

In the study developed by Paschoal and collaborators, a basis set (labeled as NMR-DKH) was developed for ^195^Pt NMR, which allowed the recovery of relativistic effects. The authors performed several NMR chemical shift calculations for many Pt(II) complexes using different theoretical methodologies. Then, a comparison was made among the three different methodologies [27].

The studied complexes were initially optimized using two different basis sets. After this step, NMR calculations were performed using NMR-DKH basis set, as follows: (PBEPBE/NMR-DKH/IEFPCM(UFF)//B3LYP/LANL2DZ/Def2-TZVPP/IEFPCM(UFF)) (Model 2) and (PBEPBE/NMR-DKH/IEFPCM(UFF)//B3LYP/LANL2DZ/Def2-SVP/IEFPCM(UFF)) (Model 3). Then, a comparison was performed using a Hamiltonian relativistic operator (ZORA), as follows: COSMO-PBE-SO-ZORA/TZ2P (Model 1) [27]. A statistical study using absolute deviation (MAD) and the relative deviation (MRD) were employed for a methodology comparison. The statistical results showed that the MAD and the MRD were 200 ppm and 6% (Model 1), 182 ppm and 6% (Model 2), and 168 ppm and 5% (Model 3), respectively [27].

As a result, the methodology chosen by Paschoal and collaborators showed an excellent agreement between the different models used. In the same way, the theoretical values obtained by relativistic calculations using NMR-DKH basis set agreed with the experimental data [27].

### 2.2. The ^195^Pt Nuclear Magnetic Resonance Chemical Shift

The importance of investigating how temperature, coordination, and chemical environment effects influence the chemical shifts is well-known. This investigation is even more important when the goal is to use this NMR parameter to propose a compound as a probe in biological systems. Keeping that in mind, the importance of geometry, chemical environment, solvent, and thermal effects for the ^195^Pt NMR chemical shift in Complex 1 (Figure 1) were analyzed. The corresponding δ(^195^Pt) values were collected and are reported in Table 1.

Analyzing the geometry effect on the ^195^Pt chemical shifts (Table 1), we observed that the δ values revealed a variation of 335.64 ppm when comparing δ_e_(PBEPBE//B3LYP) and δ_e_(PBEPBE//B3LYP/PCM(H_2_O)). Regarding the chemical shift effects for ^195^Pt, when comparing the NMR calculations with and without implicit solvent δ_e_(PBEPBE//B3LYP/PCM(H_2_O)) and δ(PBEPBE/PCM(H_2_O) //B3LYP/PCM(H_2_O)), a variation of 512.33 ppm was observed (Table 1). It is worth mentioning that this large difference in chemical shift values for different methodologies (vacuum and implicit solvent) was expected. According to the literature, the platinum chemical shift is very sensitive to geometry, electronic parameters, and chemical environments [28]. 

In this line, thermal and dynamic effects are other important contributions to be analyzed when studying chemical shifts. When the dynamic effect was included in the gas phase comparing δ_e_(PBEPBE//B3LYP) and δ^310K^(PBEPBE//ADMP)), the chemical shift value decreased by 295.51 ppm (Table 1). Moreover, when the implicit solvent effect was considered along with the dynamic effect (comparing δ_e_(PBEPBE/PCM[H_2_O]//B3LYP/PCM[H_2_O]) and δ^310K^ (PBEPBE/PCM[H_2_O]//ADMP/PCM[H_2_O])), the chemical shift value decreased by 2285.76 ppm.

Indeed, the methodology including thermal and dynamic effects with the implicit solvent was the closest approximation to real systems used in this work. In this sense, this methodology was compared to the Pt chemical shift inside the enzyme in the next topic. 

#### The ^195^Pt Chemical Shift in PI3K Enzyme Active Site

**Figure 3:**
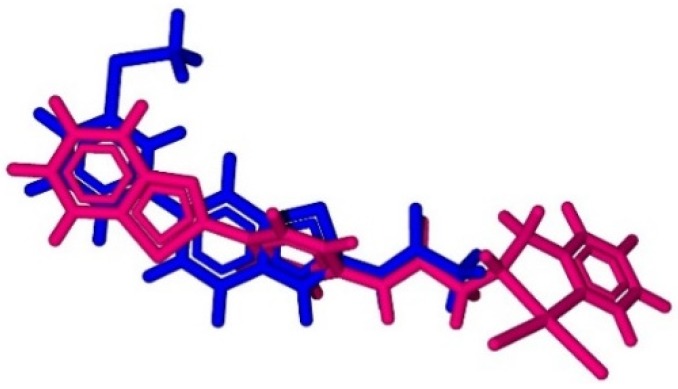
Complex 1 (pink) docked in the active site of PI3K. The active ligand is shown in blue.

Docking calculations are a fundamental tool for studies involving ligand and receptor systems [29]. In this work, a docking study was carried out to analyze the ^195^Pt chemical shift inside the enzyme PI3K, which is related to breast cancer. Then, the overlap between the complex conformations, generated during docking analysis, and the active ligand (PDB ligand) (benzothiazole derived) was performed inside the active site of the enzyme. The best superposition between the two structures is shown in Figure 3. 

**Figure 4:**
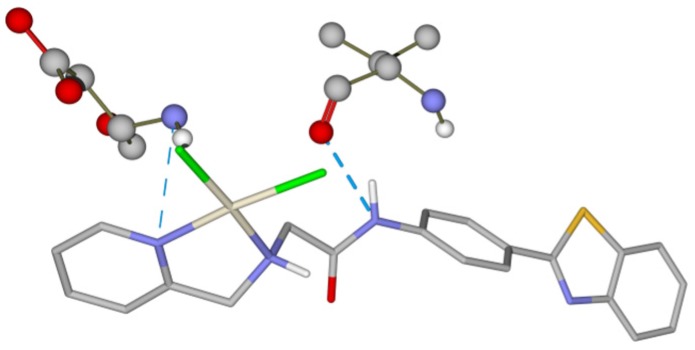
Intermolecular interaction between platinum complex, Val 882 and Asp 884.

Furthermore, the complex behavior inside of the receptor active site was also evaluated to check the stability of the complex in this chemical environment. The complex established hydrogen bonds with Val882 and Asp 884 amino acids (Figure 4). 

**Figure 5:**
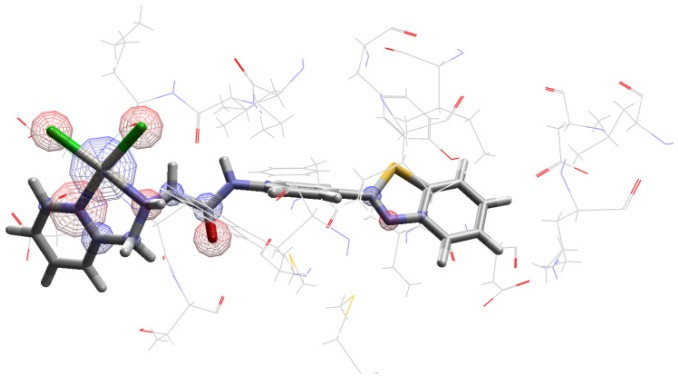
Electrostatic interactions for complex inside active site of PI3K.

Furthermore, other interactions happened to give stability to the system receptor–ligand, for example, as electrostatic and hydrophobic interactions. In this sense, the electrostatic interactions were also shown in Figure 5.

Accordingly, it can be suggested that the platinum complex is stable inside the active site of the enzyme. The platinum complex formed interactions inside the active site of enzyme and presented an intermolecular interaction energy around −150 kcal mol^−1^. To validate this methodology, a redocking procedure was performed. However, the docking study is a theoretical research and the preliminary results do not take into account pharmacokinetic proprieties. Therefore, an additional in vitro was necessary test to evaluate the interactions between this platinum complex and P13K enzyme.

To analyze the platinum complex (Figure 1) as a potential probe in biological systems, it was also necessary to perform NMR calculations for the ^195^Pt chemical shifts of the most stable complex conformation in the active site of P13K enzyme, which resulted in a δ(^195^Pt) of −1919.70 ppm. The result is in accordance to the expected value. It is well-known that the chemical environment inside the active site is hydrophobic, and the chemical shift should be close to the chemical shift obtained through the calculations without solvent δ_e_ (PBEPBE//B3LYP) for the complex (Table 1). Furthermore, the comparison between the chemical shift for the complex after docking and using thermal and dynamic and solvent effects (δ^310K^ (PBEPBE/PCM[H_2_O]//ADMP/PCM[H_2_O])) is important to understand the behavior of the complex in different environments. In this sense, the difference found when comparing these both methodologies was 2149.86 ppm.

With that in mind, these results are very interesting for this proposal. The environment inside the protein and the thermal and solvent effects are different, which show the sensitivity of the Pt chemical shift. In addition, platinum complexes are already used in medicine for cancer treatment, which may facilitate its use as a diagnosis compounds.

### 2.3. The ^195^Pt Chemical Shift for Monoaqua Platinum Complex

Since 1965, when carcinogenic properties of cisplatin were discovered, platinum (II) complexes have been used as important drugs in cancer treatment [15,16,17]. The platinum drugs present activity against cancer due their ability to bind to the N7 atom of guanine from the DNA. An important step in the interaction between platinum and DNA is the aquation of platinum complexes, which is crucial for the following binding step [18,22]. Several studies have shown that the monoaqua cisplatin complex (cis-(Pt[NH_3_]_2_[H_2_O]Cl)^+1^) is the active hydrolyzed specie at 310 K that binds to DNA, as the diaquo complex (cis-(Pt[NH_3_]_2_[H_2_O]_2_)^+2^) is the less likely specie in physiological pH [18].

In this context, it is also important to analyze the behavior of the monoaqua complex as it may be found in this way inside the cell. Thus, to propose platinum complexes as a potential probe, this aspect needs to be considered. In this sense, for monoaqua complex (Figure 2) calculations were performed including thermal and dynamic effects with an implicit solvent (δ^310K^ (PBEPBEPCM[H_2_O[//ADMP/PCM[H_2_O])). Therefore, for the study of the monoaqua compound, this methodology was employed for the evaluation of the thermal, dynamic, and solvent effects, and ^195^Pt chemical shift calculations were also performed inside the active site of the enzyme. Both results were compared to analyze the behavior of spectroscopic parameters (chemical shifts) in these different environments.

The corresponding δ(^195^Pt) value for the monoaqua platinum complex was −2813.79 ppm. Comparing this result to result for the complex before hydrolyses (Figure 1) (Table 1), a variation in the obtained ^195^Pt chemical shift values was observed. Therefore, when a water molecule was added in place of a chlorine atom, the respective value increased by 1255.79 ppm. This was expected, as it is known that platinum chemical shifts are sensitive to the coordination sphere.

In this sense, the docking study was performed to better understand the interaction of the monoaqua complex inside the active site of the enzyme. The most important moiety of the complexes for this step is the benzothiazole. The ligand inside the enzyme is derived from benzothiazole, as previously mentioned in the docking topic. From the docking studies, it was observed that the monoaquated complex established hydrogen bonds similarly to what was observed for Complex 1. The associated intermolecular interaction energy was around −140 kcal mol^−1^.

The ^195^Pt chemical shift of the most stable complex conformation in the active site of PI3K enzyme was performed for Complex 2, which resulted in a δ (195Pt) of −1783.36 ppm. The result was in accordance to the expected value and presented a big difference when compared with results for the (δ^310K^ (PBEPBEPCM[H_2_O]//ADMP/PCM[H_2_O])) methodology, which presented a value of 2813.79 ppm. Accordingly, the results for the monoaqua platinum complex (a possible specie for the platinum complex in biological system) indicated a similar behavior between this specie and the first complex. Both presented a large difference in their chemical shifts in the different environments proposed.

## 3. Methodology

### 3.1. Optimization and Molecular Dynamics (MD) Procedure

Geometries were fully optimized at the B3LYP theory level [30], with the LanL2dz [31] basis set with effective core potential (ECP) for the Pt atom and Def2-TZVPP [32] for the ligand molecule in the Gaussian 09 program [33]. These calculations were performed either in vacuum or in the presence of polarizable continuum model, using the dielectric constants of water (denoted (PCM)(H_2_O)) [14,26,27,28,29,30].

The MD simulations were performed using the atom centered density matrix propagation (ADMP) [34,35,36,37], which is a molecular dynamics model. Furthermore, the same level of theory, i.e., B3LYP/Lanl2dz, was chosen for MD. It is worth mentioning that the temperature of 310 K was included in all MD simulations to reproduce the regular biological temperature.

From the obtained MD conformations, the uncorrelated structures were selected employing the statistical inefficiency method, available in the SciLab 2.7 software [38]. This procedure was performed in order to select conformations for further NMR analysis.

### 3.2. Nuclear Magnetic Resonance (NMR) Calculations

The magnetic shielding parameters (σ) were obtained for optimized geometries, as well as for selected conformations from molecular dynamics. These calculations were performed with the gauge-including atomic orbitals (GIAO)–DFT method involving the PBEPBE functional [27,39,40,41,42,43]. These calculations used the NMRDKH basis set [26], which presented a doubly polarized triple-zeta characteristic.

The ^195^Pt NMR chemical shifts δ were calculated relative to cisplatin, which presented an experimental chemical shift (δ (^195^Pt) = −2097 ppm) [27]. Cisplatin compound was employed using the same theory level described above.

### 3.3. Molecular Docking Studies

The complexes were docked inside of the PI3K enzyme, which presented a crystallographic structure complexed with *N*-(6-)2-[methylsulfanyl]pyrimidin-4-yl)-1,3-benzothiazol-2-yl)acetamide (active ligand) (Protein Data Bank (PDB) code 3QJZ [44]; resolution = 2,90 Å) using the Molegro Virtual Docker (MVD^®^) software according to the same procedure adopted previously [14,29].

The binding site was limited to a spherical cavity with variation in the radius of 10–12 Å, and the residues was considered flexible inside a radius of 11 Å around the ligand. For these analyses, around 50 poses were obtained for each compound. Then, the analysis of the ligand–protein interactions was performed, and the overlaps were found with the active ligand inside PI3K. The best conformation of the compound was selected according to its degree of structural similarity between the active ligand and the complexes. The intermolecular interaction energy was also used for choosing the best conformation. The accommodation in the cavity was another important factor that helped evaluate the best energy of interaction with the enzyme [29,45,46].

The appropriate conformation of the studied ligand at the enzyme active site, considering some amino acids residues, was selected for the NMR analysis. Furthermore, the same theory level, i.e., PBEPBE/NMRDKH, was selected for these calculations.

## 4. Conclusions

In this research, two platinum complexes were investigated in different chemical environments (vacuum, implicit solvent, and inside active site of P13K enzyme) to propose Complex 1 (Figure 1) as a probe in biological systems. This proposal is possible because the ^195^Pt chemical shifts are very sensitive to the chemical environment. Keeping this in view, the ^195^Pt chemical shift value is a good spectroscopic parameter to propose platinum complexes as potential spectroscopic probes in biological systems. In this context, we performed chemical shift analysis of platinum-195 complexes in the above-mentioned chemical environments using a NMR-DKH basis set. The difference found in ^195^Pt chemical shifts when comparing the value in each chemical environment was expected and indicates that platinum complexes may be a potential probe in biological systems. This is possible due to the difference found when comparing the values inside and outside the enzyme. This paper proposed the use of platinum complexes not only in cancer treatments, but also in diagnosis, as these complexes have already been studied as an antitumor agent.

## Figures and Tables

**Table 1:** ^195^Pt NMR chemical shifts for Complex (Figure 1) computed at the GIAO–PBEPBE/NMR-DKH.

Level of Approximation	^195^Pt (ppm)
δ_e_(PBEPBE//B3LYP)	−1960.49
δ_e_(PBEPBE//B3LYP/PCM(H_2_O)_	−2296.13
δ_e_(PBEPBE/PCM(H_2_O)//B3LYP/PCM(H_2_O))	−1783.80
δ^310K^(PBEPBE//ADMP)	−2256.00
δ^310K^ (PBEPBE/PCM(H_2_O)//ADMP/PCM(H_2_O))	−4069.56

## Supplementary Materials

The following are available online.
