# Supplementary material for: First Attempts of the Use of 195Pt NMR of Phenylbenzothiazole Complexes as Spectroscopic Technique for the Cancer Diagnosis
Source: Molecules. 2019 Nov 2;24(21):3970. doi: 10.3390/molecules24213970 (PMC6864663; doi:10.3390/molecules24213970)
Supplement: Supplementary file 1 [file molecules-24-03970-s001.pdf]

Supplementary Material

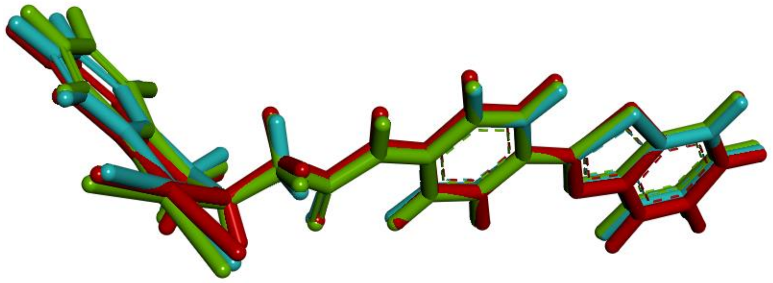

Figure S1: overlap of the three methodologies used.

Table S1: optimization calculations using relativistic and non-relativistic methods for platinum atom.

Bond lengths (Å)	Lanl2dz	aug-cc-pVTZ-pp	Zora-Def2-TZVP
Pt – Cl	2.317	2.304	2.303
Pt – Cl	2.318	2.305	2.305
Pt – N	2.047	2.037	2.034
Pt – N	2.099	2.093	2.097
**Bond angle (º)**	**Lanl2dz**	**aug-cc-pVTZ-pp**	**Zora-Def2-TZVP**
Cl – Pt – Cl	93.979	93.649	93.746
N – Pt – Cl	94.689	94.754	94.613
N – Pt – Cl	89.230	89.410	89.533
N – Pt – N	82.031	82.191	82.121

We performed optimization calculations for platinum complexes using three different methods to validate the optimization step. Based on this, the same calculations were carried out at the B3LYP/Lanl2dz, B3LYP/ aug-cc-pVTZ-pp and Zora-B3LYP/Def2-TZVP level, which is a relativistic method.

In this line, it is possible to see that when comparing the results for methodology using Lanl2dz and aug-cc-pVTZ-pp were similar to Zora-Def2-TZVP results.
